# Developing a Curriculum to Promote Professionalism for Medical Students Using Social Media: Pilot of a Workshop and Blog-Based Intervention

**DOI:** 10.2196/mededu.4886

**Published:** 2015-12-01

**Authors:** Tabor E Flickinger, Thomas O'Hagan, Margaret S Chisolm

**Affiliations:** ^1^ Division of General, Geriatric, Palliative and Hospital Medicine Department of Medicine University of Virginia School of Medicine Charlottesville, VA United States; ^2^ Department of Acute Medicine Musgrove Park Academy Musgrove Park Hospital Taunton, Somerset United Kingdom; ^3^ Department of Psychiatry and Behavioral Sciences Johns Hopkins University School of Medicine Baltimore, MD United States

**Keywords:** medical education, medical students, professionalism, social media, social networking

## Abstract

**Background:**

As the use of social media (SM) tools becomes increasingly widespread, medical trainees need guidance on applying principles of professionalism to their online behavior.

**Objective:**

To develop a curriculum to improve knowledge and skills regarding professionalism of SM use by medical students.

**Methods:**

This project was conducted in 3 phases: (1) a needs assessment was performed via a survey of medical students regarding SM use, rationale for and frequency of use, and concerns; (2) a workshop-format curriculum was designed and piloted for preclinical students to gain foundational knowledge of online professionalism; and (3) a complementary longitudinal SM-based curriculum was designed and piloted for clinical students to promote both medical humanism and professionalism.

**Results:**

A total of 72 medical students completed the survey (response rate 30%). Among the survey respondents, 71/72 (99%) reported visiting social networking sites, with 55/72 (76%) reporting daily visits. Privacy of personal information (62/72, 86%) and mixing of personal/professional identities (49/72, 68%) were the students’ most commonly endorsed concerns regarding SM use. The workshop-format curriculum was evaluated qualitatively via participant feedback. Of the 120 students who participated in the workshop, 91 completed the post workshop evaluation (response rate 76%), with 56 positive comments and 54 suggestions for improvement. The workshop was experienced by students as enjoyable, thought provoking, informative, and relevant. Suggestions for improvement included adjustments to timing, format, and content of the workshop. The SM-based curriculum was evaluated by a small-scale pilot of 11 students, randomized to the intervention group (participation in faculty-moderated blog) or the control group. Outcomes were assessed quantitatively and qualitatively via personal growth scales, participant feedback, and analysis of blog themes. There was a trend toward improvement in total personal growth scores among those students in the blog group from 3.65 (0.47) to 4.11 (0.31) (mean [SD]) with no change observed for the students in the control group (3.89 [0.11] before and after evaluation). Themes relevant to humanism and professionalism were observed in the blog discussion.

**Conclusions:**

Most medical students surveyed reported using SM and identified privacy and personal-professional boundaries as areas of concern. The workshop format and SM-based curricula were well-received by students whose formative feedback will inform the refinement and further development of efforts to promote professionalism among medical students.

## Introduction

Social media (SM) tools have become an increasingly common means of communication and are changing the flow of information in the field of medicine [1-3]. SM tools, which include Facebook, Twitter, wikis, and blogs, are Web-based technologies designed for sharing user-generated content and facilitating collaboration and networking. Most medical students today are part of the “net generation” and have grown up with these tools [4]. Surveys estimate that up to 98% of medical students have mobile phones [5] and between 60% and 70% of medical students have Facebook accounts [6-9]. Students use SM for a variety of positive purposes, such as sharing experiences and forming supportive peer networks [10]. In response to these trends, medical educators have begun to harness the potential of SM to promote active, collaborative learning [11-14].

Innovative SM curricula designed to enhance both medical humanism and professionalism are being piloted at medical schools in the United States and in other countries [15-18]. In particular, blogs have been used to combat the potential influences of the “hidden curriculum” in undermining medical students’ development of humanism and professionalism [19-21]. Through positive online interactions, medical students can develop skills to foster personal growth by reflecting upon powerful clinical experiences, encouraging introspection and sharing of information, and forming a support network of helping relationships [22-24]. SM tools can extend the benefits of reflective practices to reach trainees who may be unable to participate in in-person sessions due to the timing or geography of their training [15,16].

However, concerns have been raised regarding SM use and medical professionalism [5,25-27]. Traditional boundaries between patients and physicians, and between students and faculty, may be blurred by online interactions [28-30]. In addition, the public availability of information on patients and physicians represents a threat to privacy [31-33], with the potential for a negative impact on patient-physician relationships [34,35]. As much as 60% of medical schools responding to a survey reported incidents of medical students posting unprofessional content online [36]. Although many medical schools lack policies specific to SM use [37], guidelines are available from professional societies [38,39] and expert recommendations have been disseminated [40-42]. Little information is available on how to teach these guidelines to students and help them apply principles of professionalism to SM use. Medical educators have called for more opportunities for preventing misuse and engaging in critical analysis of SM activity [43,44]. Students have also expressed a desire for more practical recommendations and help with gray areas where there may be disagreement on what is appropriate [45].

The study’s 2 co-investigators (TEF and MSC) performed a targeted needs assessment regarding SM use among Johns Hopkins University School of Medicine first- and second-year students. Because of the concerns surrounding professionalism and SM usage, the investigators then aimed to design, pilot, and evaluate a workshop-format curriculum for students to gain knowledge relating to online professionalism. Second, the investigators aimed to design, pilot, and evaluate an SM-based curriculum to enhance overall medical student humanism and professionalism, via a focus on online behavior. Specifically, the investigators aimed for medical students to develop critical thinking regarding professionalism and SM use, gain skills of reflective practice in self-assessment and in peer interaction, and foster attitudes of empathy as a key relational aspect of professionalism.

## Methods

### Needs Assessment

The general needs assessment for this curriculum development included a systematic review of the literature regarding the use of SM in medical education [11]. The targeted needs assessment included meetings with the key stakeholders of student leaders, medical educators, and administrators. There was a perceived need for guidance on issues of online professionalism and an opportunity to address this topic during orientation activities for incoming medical students.

To gain more specific information on the targeted learners, a survey was designed for the first- and second-year medical students to assess their frequency of SM use, reasons for use, and concerns regarding use. Questions were adapted from previously published surveys [46,47]. Content validity was assessed by piloting and refining the survey with an expert focus group. The survey was administered online using SurveyMonkey; nonresponders were reminded once to complete the survey. Descriptive statistical analyses were performed, using frequencies and percentages and review of students’ free-text responses to open questions. Institutional Review Board’s approval was obtained for this study.

### Workshop-format Curriculum

#### Design

The educational methods were based on information from the needs assessment, and on *Adult Learning Theory*, which engages students in self-directed, problem-based learning with relevance to their needs and experiences, and on *collaborative learning*, which promotes learning through interaction and the construction of knowledge from experience. Best practices for SM use available from other medical centers [40] were adapted to develop cases specific to dilemmas for medical students.

The workshop was conducted as two 90-minute sessions, with 60 students each, during the required orientation sessions for the incoming first-year medical students. The medical school computer laboratory was chosen as the setting, so that each student could have a computer and the ability to share their screen with the large group via a projector. The investigators served as cofacilitators. Small groups of 8-10 students participated in activities, using their computers’ Internet search engines to locate relevant resources, and shared their findings first within their small group and then with the large group. These activities were to share examples of (1) medical students using SM to post about medical topics; (2) medical students being reprimanded for inappropriate posts on online social networks; and (3) guidelines from medical organizations regarding SM use by physicians and/or physicians-in-training. After sharing each group’s findings, all students participated in a large-group discussion about the sites that were found and issues raised. Multimedia Appendix 1 shows the outline used by the facilitators to plan the workshop sessions, including objectives, timing for activities, and resources needed.

At the end of the session, students were provided with a handout that included (1) principles of professionalism [38]; (2) guidelines for SM use [39]; and (3) selection of cases to think about guidance from best practices [40]. Multimedia Appendix 2 provides a copy of the handout.

#### Evaluation

The primary goal of the evaluation was to gain formative feedback on the pilot phase of the workshop itself, rather than a more rigorous assessment of learners. Learner satisfaction and feedback were assessed by collecting anonymous written comments at the end of each session. Qualitative analysis of the comments was performed and common themes were extracted.

### SM-Based Curriculum

#### Design

In addition to the knowledge-based workshop, an SM-based experiential curriculum was designed to address attitudes and skills, using a private, chaperoned blog. The information in the needs assessment was used to help tailor the blog to students’ level of familiarity with the technology and address students’ concerns. To investigate the usefulness of this blog, Internal Medicine clerkship students were randomized either to the intervention or control group. The intervention group was encouraged to post their own narrative reflections about powerful clinical experiences on the blog and to submit comments in response to peer- and investigator-written reflections and comments. All participant submissions were screened to ensure that all reflections and comments were Health Insurance Portability and Accountability Act compliant. The investigators served as blog moderators and posted their own reflections and comments to model medical humanism and professionalism and promote discussion. Members of the control group performed their usual clerkship without access to the study blog.

#### Evaluation

The intervention trial compared personal growth between the intervention and control conditions, measured using a questionnaire of self-reported change in 9 domains such as self-understanding, clarity of goals, values, and/or direction, and self-confidence on a 5-point Likert scale. The personal growth scale has demonstrated reliability and has been previously validated for assessment of change in Internal Medicine residents and was adapted for use in the Internal Medicine clerkship [22]. Scores on the personal growth scale survey were analyzed using *t* tests to compare mean scores in the intervention group with those in the control group.

It was hypothesized that, compared with control participants, intervention participants would have higher scores on the personal growth scale at the end of the clerkship; and that intervention participants would demonstrate an increase in personal growth between the beginning and end of the clerkship. In addition, it was hypothesized that blog posts and comments would contain themes of humanism and professionalism.

Humanism and professionalism were assessed among intervention participants by qualitative analysis of blog posts and comments exploring emerging themes. The analysis employed an inductive approach and was informed by prior qualitative analyses of medical students’ written and online reflections [15,16,48]. Institutional Review Board’s approval was obtained for the study.

## Results

### Needs Assessment

**Figure 1:**
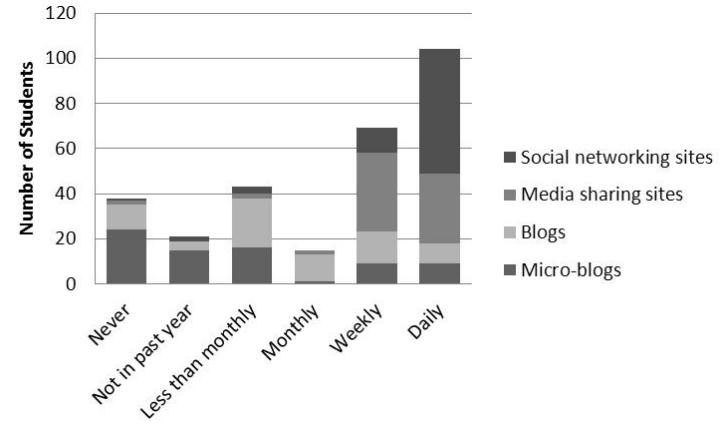
Medical students’ frequency of using SM tools.

**Figure 2:**
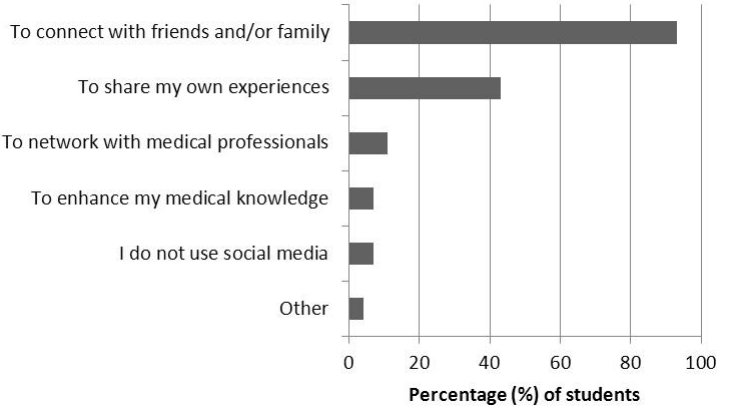
Medical students’ reasons for SM use.

**Figure 3:**
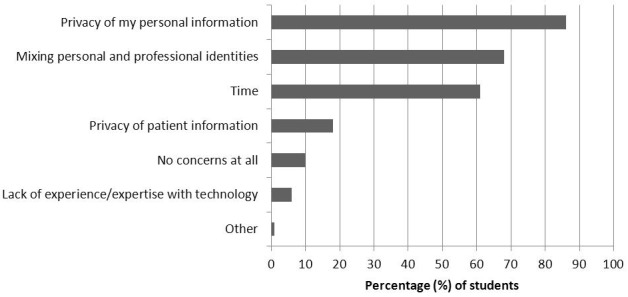
Medical students’ concerns regarding SM use.

A total of 72 students responded (30.0%, 72/240) to the survey. Figure 1 shows students’ frequency of using SM; of these, 71 respondents (71/72, 99%) visit social networking sites (eg, Facebook) and 55/72 (76%) do so daily. As much as 97% of respondents (70/72) post their own original content to social networking sites, with the majority doing this once a week. Students showed a wide range of familiarity with blogs: 11/72 (15%) had never visited a blog, 26/72 (36%) visited less than monthly, 12/72 (17%) monthly, 14/72 (19%) weekly, and 9/72 (13%) daily. A total of 28 students (28/72, 39%) had posted their own original content to a blog. Figure 2 shows students’ reasons for using SM. The most common reason cited was to connect with family and friends (67/72, 93%), followed by sharing their own experiences with others (31/72, 43%) and networking with medical professionals (8/72, 11%). Figure 3 shows students’ concerns regarding SM use. The most common concerns were privacy of personal information (62/72, 86%), mixing of personal and professional identities (49/72, 68%), time (44/72, 61%), and privacy of patient information (13/72, 18%). However, 10% of students (7/72) reported having no concerns at all.

### Workshop-Format Curriculum

Using information from the survey, the workshop was tailored to meet students’ needs. Case examples focused on the SM tools most used by students. Group discussion addressed ways to maximize the benefits of SM identified by students (such as connectivity and sharing) while avoiding risks to privacy and personal-professional boundaries.

The workshop was feasible to accomplish in the time allotted. The cofacilitators had prepared backup examples to use for each small-group activity; however, students had no difficulty in searching for relevant resources on their own and sharing them with each other. Students found examples of blogs, YouTube videos, and Twitter feeds that appeared to be posted by physicians or physicians-in-training. They also shared online news articles about examples of unprofessional behavior by medical personnel, including posting inappropriate photographs or potentially identifiable patient information. They were also able to find policy guidelines from several organizations, including the American Medical Association and Federation of State Medical Boards. In the large-group discussion, they were prompted to think critically about how to apply these guidelines to the real-world situations that their peers had found. During the discussion, facilitators emphasized critical thinking about principles of professionalism applied to each case shared by students. For example, when one participant shared a report of a medical student posting photos of a stabbing victim cared for in an emergency department rotation, facilitators prompted the group to discuss implications for patient privacy, which could be violated even if the patient’s name was not revealed. When another participant shared an article about “friending” patients on SM networks, the group reflected on the challenges of patient-clinician boundaries.

As much as 75.8% of students (91/120) submitted comments after the workshop. Categories and examples of comments are shown in Table 1. Categories were not mutually exclusive and many students provided both supportive comments and suggestions of elements they would like to be expanded or adjusted. Students found the session to be enjoyable, interesting, thought provoking, informative, and relevant. Particular aspects that they endorsed positively were the group discussion, small-group activities, and case-based format. Suggestions for improvement included adjustments to the time distribution, clarifying instructions, and providing additional resources. In particular, many students requested more discussion of challenging cases of SM use and additional practical advice on how to apply professionalism principles in these situations.

### SM-Based Curriculum

The study to evaluate the SM-based curriculum enrolled 11 participants, 8 in the blog group and 3 in the control group (using 2:1 randomization). Participants were 25.3 (2.0) years of age (mean [SD]); 8/11 (73%) were male; and 8/11 (73%) were white; 82% (9/11) were married and none had children. There were no significant demographic differences between the blog and control groups.

**Figure 4:**
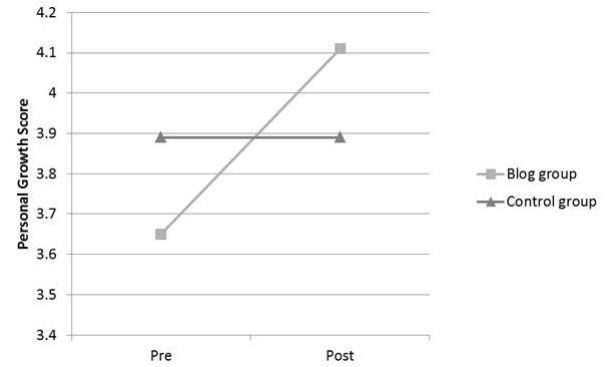
Change in medical students’ overall scores on personal growth scale, comparing blog group with control group (no blog), preclerkship and postclerkship.

Students completed the personal growth scale, which asks them to rate the extent to which they have changed on 9 items on the following scale: 1=much worse, 2=worse, 3=no change, 4=better, and 5=much better. Mean (SD) total scores for both groups were 3.72 (0.41) at baseline and 4.04 (0.26) at follow-up. As can be seen in Figure 4, there was a trend toward improvement in the blog group from 3.65 (0.47) to 4.11 (0.31) (mean [SD]) with no change in the control group 3.89 (0.11) (mean [SD]). Interestingly, the item of “more humanistic in my approach to patients” showed a trend toward improvement (mean [SD]) in the blog group (3.25 [0.71] to 4.50 [0.71]) and no change in the control group (4.00 [0.00]). These trends were consistent with the study hypotheses. However, the number of participants was too small to demonstrate significant differences.

**Table 1:** Students’ feedback on the workshop.

Themes		Examples
**Supportive comments (N=56)**		
	General comments	“Very informative. Made me think about my decisions. Really interesting and provocative. I think this is definitely something to be addressed with our generation. I really enjoyed it—eye opening.”
	Group discussion	“Wonderful discussion. Loved the ethical dilemmas addressed. Love the crowd sourcing and collaboration.”
		“It was useful to have a discussion, since I personally had not thought about these issues before.”
	Small group interactions	“I enjoyed the interactive nature of the activities. It made a topic that many might have slept through (esp. with late in afternoon timing), quite exciting.”
	Case-based format	“This was fun and a good was to meet the people in my group! I felt the review of questionable cases was helpful.”
**Constructively critical comments (N=54)**		
	Timing	“Maybe shorten the session a little bit. Keep the interactive part.”
		“I wish we had more time for the discussion at the end of the session.”
	Format	“I would have liked it better if you had examples already for us. Not sure ours was the greatest.”
		“It would have been nice to have some of the resources we found today available to us—possibly via email.”
		“Instructions at the beginning were not exactly clear.”
	Content	“Would have liked to be given more specific info regarding policy. I think it could be improved if we talked more about those “blurry” boundaries and how to negotiate with these issues”

Students randomized to the blog group also completed additional questions at follow-up. On average, students reported visiting the blog weekly to view posts or comments. All students agreed with the following statements: “I found it valuable to write my own posts/comments,” “I found it valuable to view other people’s posts/comments,” “The blog had a positive impact on my understanding of professionalism,” and “The blog had a positive impact on the professionalism of my behavior.” Strengths of the blog experience included “Ability to communicate in a format that did not depend on being in the same place at the same time,” “diversity of experience and viewpoints of contributors,” and “quick responses from faculty.” One student commented, “I’m normally not very interested in “reflecting,” but the blog format, and limiting it to only a handful of students/faculty made me more comfortable in sharing my thoughts.” The primary weakness of the blog experience was “not enough participation from other students.”

Qualitative analysis of the blog posts and comments was also performed. Themes relating to aspects of humanism included developing compassion, dealing with “difficult” patients, appreciating patients’ context, developing respect, and developing empathy for others (Table 2). For example, one post stated, “It’s relatively easy to be...respectful when it goes both ways, but not when our efforts are met with resistance or even aggression.” In the discussion, potential strategies were offered, including “I resist my impulse to think that I would behave any differently if I were in their shoes.”

Themes also related to other aspects of professionalism. Some posts related to professionalism issues specific to SM use, such as deidentification of information, student/staff privacy, and patient privacy (Table 2). Other posts concerned professionalism more broadly, such as observations of the hidden curriculum and conflict with colleagues. No breaches of professionalism occurred on the blog itself. One exchange between a student and faculty did involve feedback on further deidentification of a patient story, in which certain details might have been recognizable to someone who knew them.

**Table 2:** Qualitative analysis of the blog posts and comments relating to aspects of humanism and professionalism.

Themes		Examples
**Humanism themes**		
	Dealing with “difficult” patients	“I have no idea how to work with these patients without rolling my eyes or biting my lip.”
	Appreciating patients’ context	“Understanding the origin of the problem...can help.”
	Developing compassion	“I’ve learned that these patients require a new level of compassion.”
	Developing respect	“It’s relatively easy to be...respectful when it goes both ways, but not when our efforts are met with resistance or even aggression.”
	Developing empathy for others	“I resist my impulse to think that I would behave any differently if I were in their shoes.”
**Professionalism themes**		
	Deidentification of information	“We’re all learning how to deidentify cases.”
	Hidden curriculum	“This sort of talk is longstanding...and models a lack of collegiality.”
	Student/staff privacy	“[Are] students wanting to keep their interactions with other students private from their professors, and vice versa?”
	Patient privacy	“It is still important to protect...patient privacy.”
	Conflict with colleagues	“Do [they] truly resent the ED, or are their feelings a ‘nothing personal’ natural defense mechanism of venting?”

## Discussion

### Preliminary Findings

Most students at Johns Hopkins University School of Medicine responded to the survey report using SM at a level comparable to trainees at other institutions previously surveyed [6-9]. Respondents reported using SM for a variety of positive purposes, such as connecting with others and sharing their experiences. However, potential risks to professionalism were also expressed. They identified privacy and personal-professional boundaries as particular areas of concern. Data from the targeted needs assessment were used to develop a workshop to introduce students to professionalism guidelines in the use of SM and explored the application of these guidelines to challenging situations. Again using information from the needs assessment, an SM-based intervention was designed and piloted, in the form of a blog, to evaluate the opportunities and challenges of using SM to promote humanism and professionalism for physicians-in-training.

The piloted workshop was well-received by students, who particularly enjoyed the interactive style, case-based format, and opportunity for discussion. The workshop-based approach appears to be particularly valuable in engaging students in the topic of professionalism, which may otherwise seem too abstract for incoming trainees to discuss in a meaningful way. By emphasizing the development of critical thinking skills in applying professionalism principles to new challenges raised by SM, facilitators encouraged students to reflect on their roles as physicians-in-training and how their behavior in all aspects of their lives may reflect (positively or negatively) on their professional identities. The students provided formative feedback for the workshop, which can assist in refining future iterations of it. Later versions should include more rigorous evaluation of students’ knowledge and attitudes, to gain a better understanding of the intervention’s potential impact.

There was a trend toward increased personal growth in the intervention group and, specifically, an increase in participants’ humanistic approach to patients, which were unchanged in the control group. This study was a pilot phase with a small sample size, which does not allow definitive conclusions of effectiveness. However, these preliminary data are promising. It is possible that future expansions of this curriculum could be used to combat the decline in empathy observed in prior studies of medical students during their clerkship training [49].

Further implementation on a larger scale will be needed before any firm conclusions can be drawn. However, the goal will be to allow students to apply the knowledge gained from the workshop while developing skills in using SM and, ultimately, promote medical humanism and professionalism. As with any new technology, it is important to weigh the risks versus benefits in incorporating SM tools into medical education. No breaches of professionalism were observed on the faculty-moderated blog; however, expansion of the intervention does carry a potential risk, particularly in violations of patient privacy. This pilot blog was accessible only to faculty moderators and study participants, to ensure that any possible inappropriate posts would not be public and could be removed. The moderators also worked closely with IT security consultants at their institution to minimize potential risks. Allowing students to practice using SM in a professional manner within a safe environment shows promise as a teaching method that balances the risks and benefits of SM use.

### Limitations

This work has several limitations. For the needs assessment, there was a relatively low response rate. Although this is not atypical of survey studies of medical trainees’ online behavior [50], there may have been some selection bias if the students who responded had different attitudes and behaviors regarding SM than those who did not respond. Students’ SM use outside of the workshop setting was not observed. Therefore, it is not possible to say whether their use of SM was changed by the intervention. For the SM intervention, the major limitation was the small number of participants, which meant statistically significant differences were not observed. Recruitment was a challenge in terms of integrating the intervention with students’ other activities. Blog participation was also affected by technological problems. This issue could be addressed by using a different platform for future blog interventions with links through systems that students already sign into (eg, blackboard). For both the workshop and SM-based curricula, it is difficult to say if incidents of unprofessional behavior have been prevented, when the baseline rate is rare and not precisely known.

### Conclusions

This paper contributes an intervention that was useful at one institution, and could be adapted to others as well, to address emerging issues of professionalism and SM use among medical students. Modern medical students have grown up with SM and may take its use for granted, without fully realizing the implications of their online behavior in their new roles as physicians-in-training. Medical educators have an opportunity not only to provide valuable guidance to students in using SM wisely, but also to promote the development of professional identities by implementing SM interventions into the medical curricula.

## Appendix

Multimedia Appendix 1 Workshop outline for facilitators.

Multimedia Appendix 2 Workshop handouts for students.

## Abbreviations

SM: social media
