# Supplementary material for: Developing a Curriculum to Promote Professionalism for Medical Students Using Social Media: Pilot of a Workshop and Blog-Based Intervention
Source: JMIR Med Educ. 2015 Dec 1;1(2):e17. doi: 10.2196/mededu.4886 (PMC5041365; doi:10.2196/mededu.4886)
Supplement: Multimedia Appendix 1 [file mededu_v1i2e17_app1.pdf]

Professionalism and Social Media for Medical Students

Goals
1. Generally, introduce medical students to new role as trainees in medical profession
2. Specifically, give students guidance in avoiding unprofessional social media use

Setting: JHU SOM orientation, 2 sessions of 1 hour each
Subjects: Incoming first-year medical students, 2 groups of 60 each

Objectives
At the end of the workshop, all students will be able to:
1. Identify guidelines for professionalism in social media use
2. Apply guidelines to cases of social media use by medical students

Opening presentation: large group [10 mins]
1. Introduce objectives
2. Give instructions for small-group activity

Activity: small groups [10 mins]
Separate into 3 groups of about 20 by tables, to spend a few minutes searching for examples of:
1. Medical students using social media (eg. YouTube, blog, Facebook, Twitter) to post about medical topics
2. Medical students getting in trouble for inappropriate posts
3. Guidelines from medical organization regarding social media use by physicians and/or physicians-in-training

Discussion: large group [40 mins]
1. Each table shares examples they found
2. If particular principle or case type not found, can use our examples

Handout to take away
1. Principles of professionalism
2. Guidelines for social media use
3. Selection of cases to think about

Resources
1. Room for students
2. Two facilitators (us)
3. Computers for students to use
4. Handouts, cards

Evaluation
1. How well do students participate in discussion? [Assess by observation]
2. Do students have suggestions for improving the workshop? [Assess by anonymous suggestions submitted on index cards]
