# Supplementary material for: Developing a Curriculum to Promote Professionalism for Medical Students Using Social Media: Pilot of a Workshop and Blog-Based Intervention
Source: JMIR Med Educ. 2015 Dec 1;1(2):e17. doi: 10.2196/mededu.4886 (PMC5041365; doi:10.2196/mededu.4886)
Supplement: Multimedia Appendix 2 [file mededu_v1i2e17_app2.pdf]

AMA Policy: Professionalism in the Use of Social Media

The Internet has created the ability for medical students and physicians to communicate and share information quickly and to reach millions of people easily. Participating in social networking and other similar Internet opportunities can support physicians' personal expression, enable individual physicians to have a professional presence online, foster collegiality and camaraderie within the profession, provide opportunity to widely disseminate public health messages and other health communication. Social networks, blogs, and other forms of communication online also create new challenges to the patient-physician relationship.

Physicians should weigh a number of considerations when maintaining a presence online:

(a) Physicians should be cognizant of standards of patient privacy and confidentiality that must be maintained in all environments, including online, and must refrain from posting identifiable patient information online.

(b) When using the Internet for social networking, physicians should use privacy settings to safeguard personal information and content to the extent possible, but should realize that privacy settings are not absolute and that once on the Internet, content is likely there permanently. Thus, physicians should routinely monitor their own Internet presence to ensure that the personal and professional information on their own sites and, to the extent possible, content posted about them by others, is accurate and appropriate.

(c) If they interact with patients on the Internet, physicians must maintain appropriate boundaries of the patient-physician relationship in accordance with professional ethical guidelines just, as they would in any other context.

(d) To maintain appropriate professional boundaries physicians should consider separating personal and professional content online.

(e) When physicians see content posted by colleagues that appears unprofessional they have a responsibility to bring that content to the attention of the individual, so that he or she can remove it and/or take other appropriate actions. If the behavior significantly violates professional norms and the individual does not take appropriate action to resolve the situation, the physician should report the matter to appropriate authorities.

(f) Physicians must recognize that actions online and content posted may negatively affect their reputations among patients and colleagues, may have consequences for their medical careers (particularly for physicians-in-training and medical students), and can undermine public trust in the medical profession.

"AMA Policy: Professionalism in the Use of Social Media." American Medical Association, 2012 Annual Meeting. http://www.ama-assn.org/ama/pub/meeting/professionalism-social-media.shtml

Practical Suggestions for Best Practices

1. **Take Responsibility and Use Good Judgment.** You are responsible for the material you post on personal blogs or other social media. Be courteous, respectful, and thoughtful about how other Personnel may perceive or be affected by postings. Incomplete, inaccurate, inappropriate, threatening, harassing or poorly worded postings may be harmful to others. They may damage relationships, undermine your institution's brand or reputation, discourage teamwork, and negatively impact the institution's commitment to patient care, education, research, and community service.

2. **Think Before You Post.** Anything you post is highly likely to be permanently connected to you and your reputation through Internet and email archives. Future employers can often have access to this information and may use it to evaluate you. Take great care and be thoughtful before placing your identifiable comments in the public domain.

3. **Protect Patient Privacy.** Disclosing information about patients without written permission, including photographs or potentially identifiable information, is strictly prohibited. These rules also apply to deceased patients and to posts in the secure section of your Facebook page that is accessible by approved friends only.

4. **Protect Your Own Privacy.** Make sure you understand how the privacy policies and security features work on the sites where you are posting material.

5. **Respect Work Commitments.** Ensure that your blogging, social networking, and other external media activities do not interfere with your work commitments.

6. **Identify Yourself.** If you communicate in social media about your institution, disclose your connection and your role. Use good judgment and strive for accuracy in your communications. False and unsubstantiated claims, and inaccurate or inflammatory postings may create liability.

7. **Use a Disclaimer.** Where your connection to your institution is apparent, make it clear that you are speaking for yourself and not on behalf of any organization. A disclaimer, such as, "The views expressed on this [blog; website] are my own and do not reflect the views of my employer," may be appropriate.

8. **Respect Copyright and Fair Use Laws.** For your institution's protection as well as your own, it is critical that you show proper respect for the laws governing copyright and fair use of copyrighted material owned by others, including your institution's own copyrights and brands.

9. **Protect Proprietary Information.** Do not share confidential or proprietary information that may compromise business practices or security. Similarly, do not share information in violation of any laws or regulations.

10. **Seek Expert Guidance.** Consult with the Marketing & Communications Department if you have any questions about the appropriateness of materials you plan to publish or if you require clarification on whether specific information has been publicly disclosed before you disclose it publicly.

Adapted from: "Mount Sinai Medical Center Social Media Guideline." Mount Sinai School of Medicine. http://www.mssm.edu/about-us/services-and-resources/faculty-resources/handbooks-and-policies/faculty-handbook/institutional-policies/social-media-guidelines

Example Scenarios of Professionalism and Social Media Use

1. A medical student receives a “friend” request on his Facebook page from a patient encountered during his clinical skills course.

2. A medical student has a blog on which she posts reflections about both personal and professional issues. She has just finished her clinical skills course. A patient, whom she met during the course, comments on the student’s blog and discloses protected health information with the expectation that the student will continue the discussion.

3. A medical student is on his outpatient clerkship. He “tweets” that he just finished seeing an interesting patient with his preceptor and describes the clinical findings of that patient.

4. A medical student is shadowing an OBYGYN physician. She posts (on her Facebook page) a picture of a baby whose delivery she observed, expressing joy, best wishes to the family, and congratulating everyone involved in this excellent patient outcome.

5. A medical student writes in her blog, naming an attending physician who did minimal teaching and recommending that other students not take clinical electives with that physician.

6. A medical student on a research elective blogs that the laboratory equipment he is using should have been replaced years ago and is unreliable.

7. A medical student wearing a Johns Hopkins tee-shirt is tagged in a photo taken at a local bar and posted on a friend’s Facebook page. The medical student is clearly inebriated.

8. A medical student uses an alias and blogs that Johns Hopkins has the lowest bone marrow transplantation complication rate in the world.

9. A medical student creates a social media website to share and discuss both pre-clinical and clinical medical knowledge (e.g., "Cardiology Interest Group" on Facebook).

Adapted from: “Mount Sinai Medical Center Social Media Guideline.” Mount Sinai School of Medicine. http://www.mssm.edu/about-us/services-and-resources/faculty-resources/handbooks-and-policies/faculty-handbook/institutional-policies/social-media-guidelines

Relevant Guidelines for Example Scenarios

1. It is almost always inappropriate to accept "friend" request from patients, unless the doctor-patient relationship has ended. Even after the doctor-patient relationship has ended, it would be inappropriate to discuss health-related information. Best practices: Protect patient privacy.

2. Social media discussion with a patient should not directly address health concerns of individual patients. Best practices: Protect patient privacy.

3. It is difficult to be certain that information disclosed in posts (such as on Twitter®) is not identifiable to that particular patient. The best type of posting would include very general information. Other posts by the same student could indicate his/her medical school and current rotation, leading to circumstances that indirectly identify the patient, such as by naming a very rare disease. Best practices: Protect patient privacy.

4. Without written patient/representative consent, this is a clear violation of patient confidentiality, even if the patient is not named. Best practices: Protect patient privacy.

5. Legitimate critique of an educational activity is appropriate, so long as professionalism is maintained. There are more effective and less public mechanisms for relaying this type of information. Best practices: Use good judgment; think before you post.

6. The public disclosure of negative information increases the liability for the Medical Center and is clearly unprofessional. There are legitimate and confidential mechanisms for improving quality at the Medical Center. Best practices: Use good judgment; think before you post.

7. The two issues are that: (1) the logo identifies the affiliation to the institution; and (2) the unprofessional behavior of the student is available for all to see, including future employers and patients. The medical student did not post the photo, but should do everything possible to have the photo removed and remove the tagging link to the student's own Facebook page. Best practices: Protect your own privacy; think before you post.

8. This may be a violation of Federal Trade Commission regulations that prohibit false or unsubstantiated claims, and does not disclose the employee's material relationship to the institution. Best practices: Identify yourself; protect proprietary information.

9. This is a learning community environment, in which medical knowledge is exchanged, shared and discussed. While the goal is laudable, there are still risks. A disclaimer is necessary, since postings may be incorrect, taken out of context, or improperly referenced. The moderator should take precautions to prevent the posting of information or photographs that are potentially identifiable to a particular patient. Best practices: Protect patient privacy; identify yourself; use a disclaimer.
